# Predictive value of comb-push ultrasound shear elastography for the differentiation of reactive and metastatic axillary lymph nodes: A preliminary investigation

**DOI:** 10.1371/journal.pone.0226994

**Published:** 2020-01-13

**Authors:** Adriana Gregory, Max Denis, Mahdi Bayat, Viksit Kumar, Bae Hyung Kim, Jeremy Webb, Rohit Nayak, Saba Adabi, Duane D. Meixner, Eric C. Polley, Robert T. Fazzio, Mostafa Fatemi, Azra Alizad

**Affiliations:** 1 Department of Radiology, Mayo Clinic College of Medicine, Rochester, Minnesota, United States of America; 2 Department of Physiology and Biomedical Engineering, Mayo Clinic College of Medicine,Rochester, Minnesota, United States of America; 3 Biomedical Statistics and Informatics, Mayo Clinic College of Medicine, Rochester, Minnesota, United States of America; Gangnam Severance Hospital, Yonsei University College of Medicine, REPUBLIC OF KOREA

## Abstract

**Objectives:**

To evaluate the predictive performance of comb-push ultrasound shear elastography for the differentiation of reactive and metastatic axillary lymph nodes.

**Methods:**

From June 2014 through September 2018, 114 female volunteers (mean age 58.1±13.3 years; range 28–88 years) with enlarged axillary lymph nodes identified by palpation or clinical imaging were prospectively enrolled in the study. Mean, standard deviation and maximum shear wave elastography parameters from 117 lymph nodes were obtained and compared to fine needle aspiration biopsy results. Mann-Whitney U test and ROC curve analysis were performed.

**Results:**

The axillary lymph nodes were classified as reactive or metastatic based on the fine needle aspiration outcomes. A statistically significant difference between reactive and metastatic axillary lymph nodes was observed based on comb-push ultrasound shear elastography (CUSE) results (p<0.0001) from mean and maximum elasticity values. Mean elasticity showed the best separation with a ROC analysis resulting in 90.5% sensitivity, 94.4% specificity, 0.97 area under the curve, 95% positive predictive value, and 89.5% negative predictive value with a 30.2-kPa threshold.

**Conclusions:**

CUSE provided a quantifiable parameter that can be used for the assessment of enlarged axillary lymph nodes to differentiate between reactive and metastatic processes.

## Introduction

Metastatic involvement of axillary lymph nodes (ALNs) in patients with breast cancer is an important prognostic factor used for cancer staging and to determine the best treatment option [1–3]. Given that more than 75% of the lymphatic drainage from the breast passes through the ALNs, metastatic ALN involvement is prevalent [4].

ALNs involved in the metastatic process have morphological changes that can be detected by palpation (i.e., increase in size) or through clinical imaging. Thus, ultrasonography of the axilla is routinely performed in a patient with suspected breast cancer. Several sonographic features can be detected including an increase in size, increase in cortical thickness, change in shape, lobulation of the cortex, displacement or replacement of the fatty hilum, etc. [5, 6]. These features can provide valuable information for the characterization of abnormal ALNs [7–9], and help identify lymph nodes suitable for needle biopsy [10–17].

Not all abnormal ALN’s are caused by breast cancer metastasis; other processes, such as infection, can lead to similar morphological changes observed under ultrasound (US) B-mode imaging. Although these nodes are benign (i.e., reactive ALNs), based on US characteristics, they often undergo needle biopsy [18]. However, several differences can be observed under a histopathological examination. In the case of reactive ALNs, the increase in cortex size is usually due to a regional injury or infection which increases the number and size of follicular centers [19]. On the other hand, an increase in cortex size in metastatic ALNs is due to the deposition of cancer cells that travel from the malignant breast mass. Once cancer cells are in the ALN, there is an increase of the collagen fiber density [20]. Collagen is a very stiff material with an elastic modulus of 37.7 to 42.4-GPa [21], which may change the mechanical properties of metastatic ALNs. Normal ALNs have been reported to have an elastic modulus of 2.4 to 38.9-kPa [13, 16].

Ultrasound shear wave elastography (SWE) noninvasively provides additional information useful for the differentiation of tissues with different elastic properties. The most well-known SWE techniques are Supersonic Imagine (SSI) and Virtual touch tissue quantification (VTTQ). SSI excites tissue by generating three push beams at different focal points along the beam axis at a supersonic speed which creates two shear waves that propagate in opposite directions [22]. VTTQ uses a single push to excite the tissue and analyzes the displacement at multiples time points [23]. For this study we used Comb-push ultrasound shear elastography (CUSE), a type of SWE that uses four push beams laterally distributed to create multiple shear waves in tissues. Local shear wave speed is measured at each pixel by averaging the speed of left-to-right and right-to-left waves. Finally, the estimated speeds can be translated into an elasticity map [24, 25].

In this study, B-mode US features and elasticity parameters were assessed to evaluate the predictive performance of CUSE for the differentiation of reactive and metastatic axillary lymph nodes. Although, other SWE techniques such as Supersonic Imagine [13, 14, 16, 26] and Virtual tissue quantification [15, 27] have been used to predict ALN metastasis; to the best of our knowledge, this is the first report showing the performance of CUSE for the differentiation of reactive and metastatic ALNs.

## Materials and methods

This prospective study received institutional review board approval, Mayo Clinic IRB#: 13–006035, and was Health Insurance Portability and Accountability Act compliant. A signed written informed consent was obtained from all participants prior to the study.

### Study population

From June 2014 to September 2018, 125 women aged 18 years and older who were scheduled for US guided ALN fine needle aspiration (FNA) biopsy as part of their clinical care plan, and did not receive chemotherapy, were recruited for the study. Patients who did not undergo biopsy (n = 5) and patients with no lymphoid tissue identified after FNA biopsy (n = 6) were excluded. The remaining 114 patients (28–88 years old, mean age 58.1 years) were included for data analysis. Breast masses were present in 110 (96.5%) patients. From this group, 26 (23.6%) were known to have malignant breast masses prior to the study; however, the status of the ALNs was unknown. Three patients (2.6%) had incidental findings in the axillary area under clinical imaging (ie, screening mammography, magnetic resonance imaging (MRI) or single photon emission computed tomography / computed tomography scan), and one patient (0.9%) presented with a palpable axillary lump. In total, 117 lymph nodes (3 patients had 2 abnormal ALNs) were examined with clinical US, CUSE and ultrasound-guided FNA biopsy.

### Clinical ultrasound features

Morphological characteristics observed under clinical ultrasound were measured and evaluated. They included the node shape (ratio of long and short axis distance), cortical thickness, presence or absence of a fatty hilum, and symmetry of the cortex (i.e., symmetric or asymmetric).

### Imaging protocol: comb-push ultrasound shear elastography

The study was performed with a GE LOGIQ E9 US machine with CUSE implementation using a linear array transducer 9L-D (frequency range: 2–8 MHz) (GE Healthcare). CUSE is a 2D SWE technique that uses four laterally distributed equidistant push beams to induce shear waves in tissues [25]. Ultrasound and CUSE images were acquired by an expert sonographer with 30 years of experience in US imaging and more than 6 years of experience in SWE imaging. A sequence of one B-mode image followed by 5 SWE images was obtained per participant at the cross-section showing the largest cortex enlargement. Each SWE image was captured by default with a corresponding B-mode image. During each acquisition the patient was asked to stop breathing for 3 seconds to prevent motion artifacts. Minimal compression was applied with the transducer to avoid pre-compression effects.

After all images were acquired, shear wave speed was immediately calculated by using the 2D dual circle tool from the scanner, which allows visualization of the selected region of interest (ROI) on the B-mode image and the shear wave map simultaneously. Circular ROIs 3mm in diameter were placed on the lymph node enlarged cortex area (up to 3 non-overlapping ROIs depending on the size of the area). When more than one ROI was placed, the final mean and maximum elasticity were obtained by calculating the average from all mean values and the maximum from all maximum values. The average of mean, standard deviation (SD) and maximum measurements from at least three shear wave speed maps were calculated and converted to mean (E_mean_), SD (E_SD_) and maximum (E_max_) elasticity values in terms of Young’s modulus, respectively. The young’s modulus values are calculated from the following expression,

$$E=3\rho e^{3}$$ (1)

where *ρ* = 1000-Kg/m^3^represents the tissue density and *c* is the shear wave speed.

### Fine needle aspiration biopsy

FNA biopsy was performed approximately one hour after the CUSE test. Using ultrasound guidance and standard sterile technique, a 25-gauge needle was used by one of our board-certified radiologists to obtain six fine needle aspirates for each case. Slides were prepared immediately and sent for cytology. Citologic diagnosis was made by a cytologist with more than 15 years of experience. Specimens with no lymphoid tissue on cytological examination of showing were excluded (6 patients); only ALNs with positive or negative for malignancy cytology results were included for data analysis.

### Statistical methods

Statistical analyses were performed by using the software MedCalc (MedCalc Software bvba, Ver. 17.5.5, 2017) and JMP (SAS Institute Ver. 13.0.0, 2016). Mean, SD and maximum elasticity values from each ALN were matched to FNA biopsy cytologic results and evaluated by a receiver operating characteristic (ROC) curve analysis. Area under the curve, sensitivity, specificity, positive predictive values, negative predictive values, and 95% confidence intervals (CI) were calculated. Optimal cutoff values were determined at maximum sensitivity and specificity. A Mann-Whitney U test was performed to compare the elasticity values of reactive and metastatic ALNs. Statistical significant difference were considered at *p*<0.05.

## Results

### Cytology findings

Cytologic examination from the FNA biopsies revealed that 54 (46%) lymph nodes (53 patients) were reactive and 63 (54%) lymph nodes (61 patients) had metastasis from breast cancer. From the reactive group, 12 patients were diagnosed with benign breast masses and 3 with only lymphadenopathy; further testing was therefore not warranted in these individuals. The remaining 38 patients were diagnosed with malignant breast masses. From this group, 29 underwent sentinel lymph node (SLN) excision with negative results.

### Sonographic findings

The average of the long axis distance for the 117 ALNs was 15.9 mm (range 5.5–36 mm), while the mean short axis measurement was 8.4 mm (range 3.2–24 mm). No statistical difference (p = 0.087) was found between the long axis average of the reactive (14.7±6 mm) vs. the metastatic (16.9±7mm) ALNs. The mean short axis measure of metastatic ALNs (9.2±3mm), though, was statistically greater (p = 0.0067) than that of the reactive ALNs (7.5±3 mm). The ratio of long and short axis values was not significantly different (p = 0.33) between reactive (2.1±0.8) and metastatic (1.9±0.6) ALNs.

The mean cortical thickness of the 117 ALNs was 6.4 mm (range 3.1–25 mm). The mean cortical thickness of reactive ALNs (4.9±2 mm) was significantly smaller (p<0.0001) than the mean cortical thickness of metastatic ALNs (7.8±4 mm). Asymmetric cortical lobulation was found in 89 ALNs (54 were metastatic, 35 were reactive), while 28 ALNs were symmetric (9 were metastatic, 19 were reactive). Twenty-five ALNs were completely hypoechoic with no hilum (21 were metastatic, 4 were reactive), and 92 ALNs had a hyperechoic hilum (42 were metastatic, 50 were reactive).

### CUSE results

**Fig 1:**
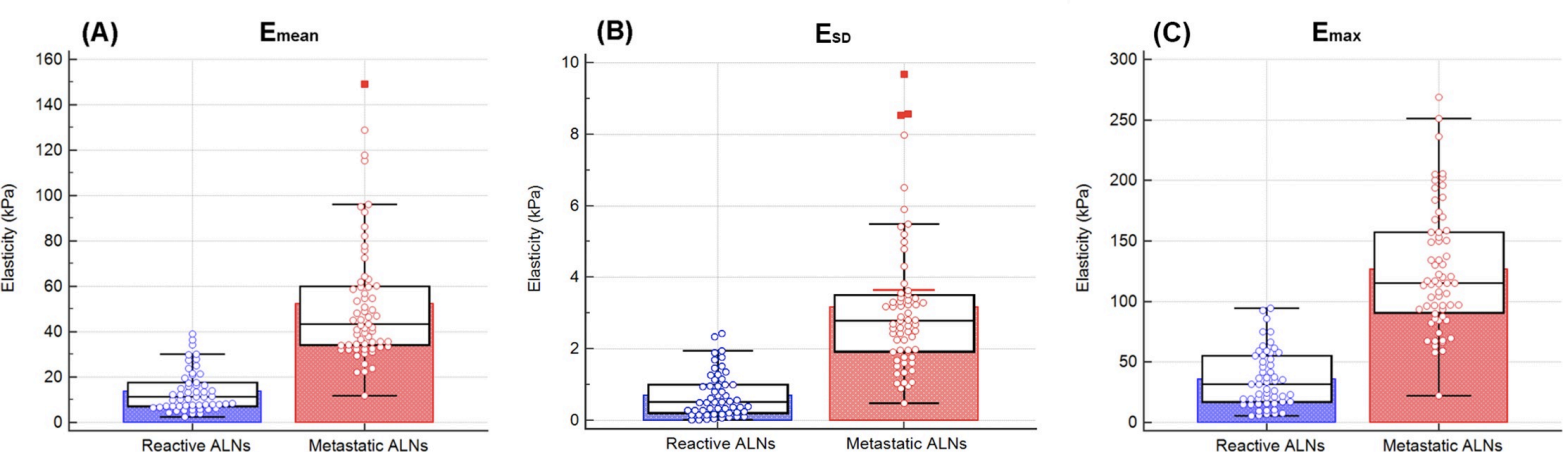
Data distribution plots. (A) E_mean_ (B) E_SD_ and (C) E_max_ distributions. The box depicts the median, upper and lower quartiles. The whiskers indicate the maximum and minimum nonextreme values. The bars represent the mean values. Outliers are indicated by ■.

A statistically significant difference (*p*<0.0001) between reactive and metastatic ALNs based on Young’s modulus was observed. Fig 1 illustrates the data distribution for mean, SD and maximum elasticity values. The mean elasticity (E_mean_) acquired with CUSE ranged from 2.4 to 149.2 kPa, with an average value of 33.7±29 kPa (CI: 28.4–38.9); E_SD_ ranged from 0.02 to 9.7 kPa, with an average value of 2.03±1.9 kPa (CI: 1.7–2.4); finally, the maximum elasticity (E_max_) ranged from 5.3 to 268.7kPa, with an average value of 82.6±61.4 kPa (CI: 71.3–93.8). Further summary of the distributions based on pathology are shown in Table 1.

**Table 1:** Summary of CUSE measurements in reactive and metastatic axillary lymph nodes.

	Reactive	Metastatic	p-value
E_mean_	(11.3) 13.8±9.1	(43.4) 52.3±27.3	<0.0001
E_max_	(31.8) 35.9±23.5	(115.3) 126.6±51.2	<0.0001
E_SD_	(0.51) 0.7±3.2	(2.79) 3.2±1.9	<0.0001

Numbers in parenthesis are the median followed by mean ± standard deviation

### Diagnostic performance of CUSE and B-mode US Imaging

ROC analysis of ultrasonography and mean, SD and maximum elasticity values are summarized in Table 2. The E_mean_ and E_max_ proved to be good predictors for malignancy showing that metastatic ALNs have higher elasticity values than reactive ALNs. E_SD_ was the parameter with the third best result and showed that reactive ALNs present a more homogeneous elasticity map (i.e. less variance) compared to metastatic ALNs. On the other hand, ultrasonography features did not show high accuracy, since morphological features of reactive and metastatic lymph nodes presented similarly. The best ultrasonography feature for differentiating metastatic and reactive ALNs was the cortical thickness measurement; a cutoff of >5.4 mm resulted in 70.1% accuracy in differentiating reactive (cortically thinner) from metastatic (cortically thicker) nodes.

**Table 2:** Performance of conventional ultrasonography and CUSE in the assessment of reactive and metastatic ALNs.

	Sensitivity	Specificity	AUC	Positive Predictive Value	Negative Predictive Value	Accuracy	Optimal threshold
**Ultrasonography**							
**Long axis**	28.6 (18/63) [17.9–41.3]	90.7 (49/54) [79.7–96.9]	0.59	78.3 (18/23)	52.1 (49/94)	57.3 (67/117)	>21 mm
**Short axis**	71.4 (45/63) [58.7–82.1]	57.4 (31/54) [43.2–70.8]	0.65	66.2 (45/68)	63.3 (31/49)	65 (76/117)	>6.8 mm
**Long/Short axis ratio**	82.5 (52/63) [70.9–90.9]	29.6 (16/54) [18–43.6]	0.55	57.8 (52/90)	59.3 (16/27)	58.1 (68/117)	≤2.4
**Cortical thickness**	61.9 (39/63) [48.8–73.9]	79.6 (43/54) [66.5–89.4]	0.75	78 (39/50)	64.2 (43/67)	70.1 (82/117)	>5.4 mm
**Hilum**	33.3 (21/63) [22–46.3]	92.6 (50/54) [82.1–97.9]	0.63	84 (21/25)	54.3 (50/92)	60.7 (71/117)	
**Cortex symmetry**	85.7 (54/63) [74.6–93.3]	35.2 (19/54) [22.7–49.4]	0.60	60.7 (54/89)	67.9 (19/28)	62.4 (73/117)	
**CUSE**							
**E**_**mean**_	90.5 (57/63) [80–96]	94.4 (51/54) [84.6–98.8]	0.97	95 (57/60)	89.5 (51/57)	92.3 (108/117)	>30.2 kPa
**E**_**max**_	92.1 (58/63) [82.4–97.4]	90.7 (49/54) [79.7–96.9]	0.97	92.1 (58/63)	90.7 (49/54)	91.5 (107/117)	>66.5 kPa
**E**_**SD**_	90.5 (57/63) [80.4–96.4]	83.3 (45/54) [70.7–92.1]	0.95	86.4 (57/66)	88.2 (45/51)	87.2 (102/117)	>1.3

Numbers in parentheses are the raw data. Data in brackets are the 95% confidence intervals.

**Fig 2:**
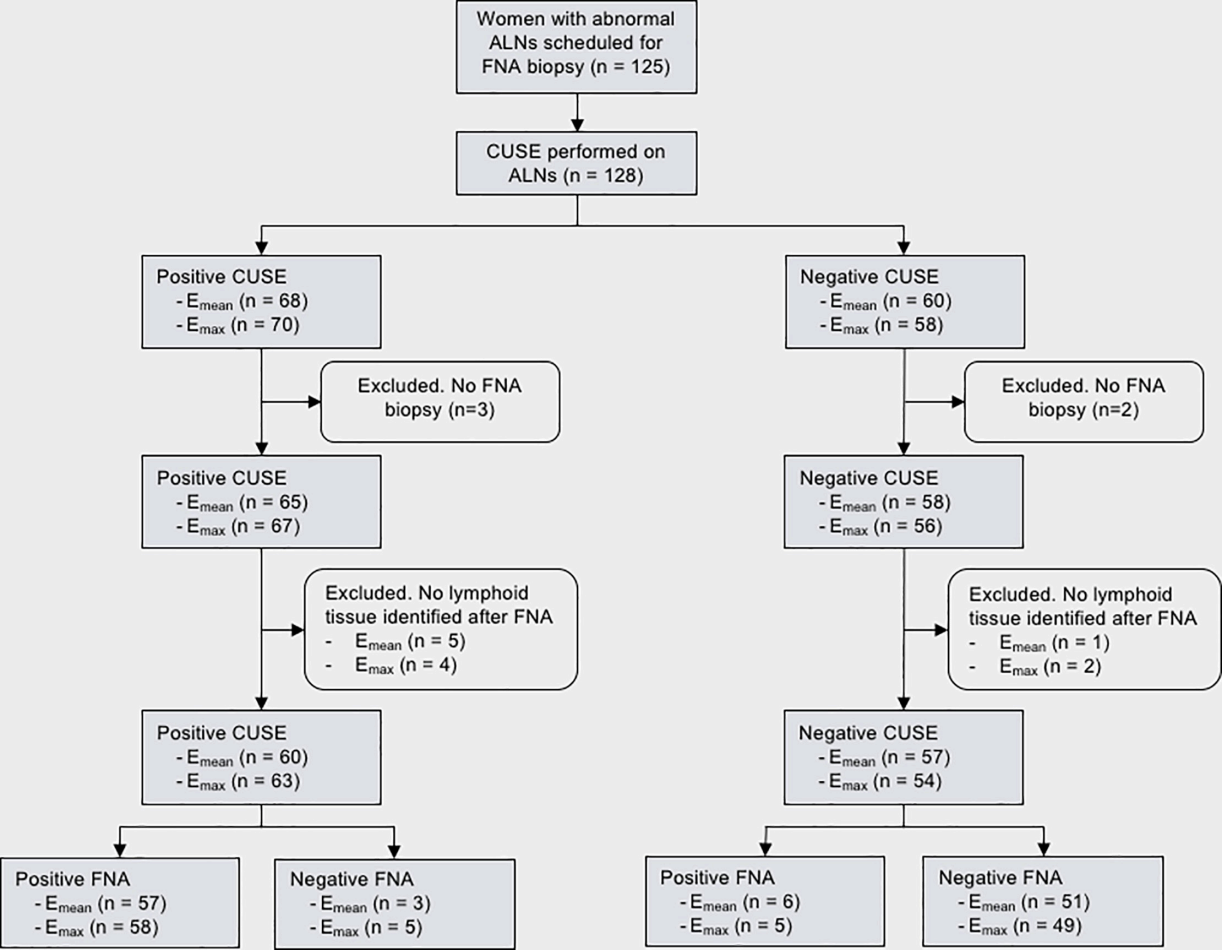
Flowchart of the study population showing CUSE and FNA biopsy results.

A total of 6 false positive cases resulted from E_mean_ (n = 3) and E_max_ (n = 5) with a 6.2-mm average cortical thickness, 5 ALNs showed asymmetric cortex and 1 ALN had absent fatty hilum. A total of 8 false negative cases resulted from E_mean_ (n = 6) and E_max_ (n = 5) with a 4.8-mm average cortical thickness, 5 ALNs had a symmetric cortex and 5 ALNs presented a fatty hilum (Fig 2).

**Fig 3:**
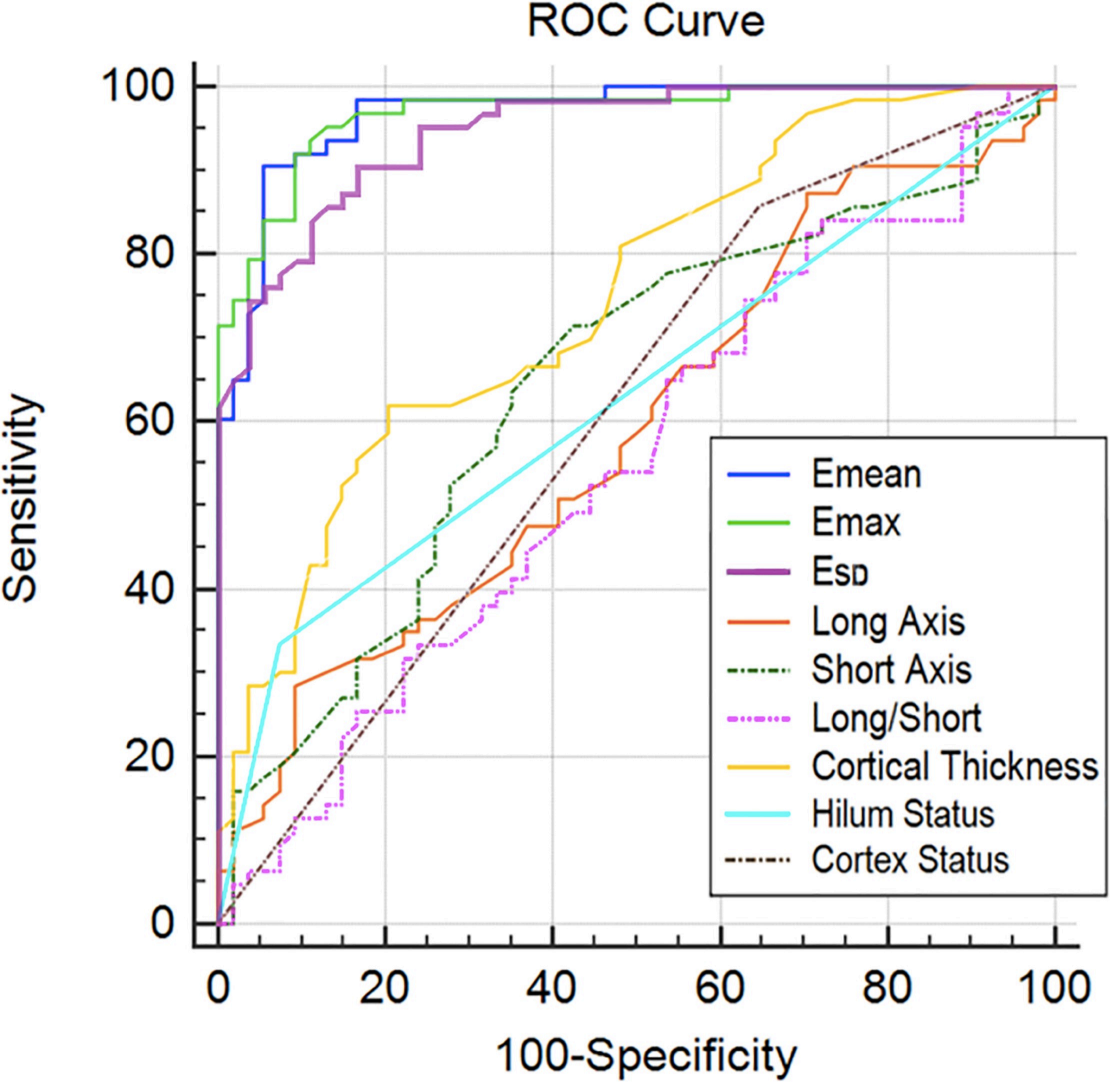
Receiver operating characteristic (ROC) curves of conventional ultrasound features and CUSE elasticity parameters.

In Fig 3, the differences of the ROCs for B-mode features and elastography parameters can be observed. Substantial overlap exists between the E_mean_ and E_max_ ROC areas. Of all the characteristics plotted, these two show the highest overall sensitivity and specificity, since their curves closely follow the left hand border (indicating high specificity) and top border (indicating high sensitivity) of the ROC space. A comparison analysis showed no significant difference between the two (p = 0.9641). Finally, a statistically significant difference was found between the ROC areas of the elasticity parameters (i.e., E_mean_ and E_max_) and that of the best conventional ultrasound predictor (i.e., cortical thickness) with a p-value of p<0.0001 in both cases.

**Fig 4:**
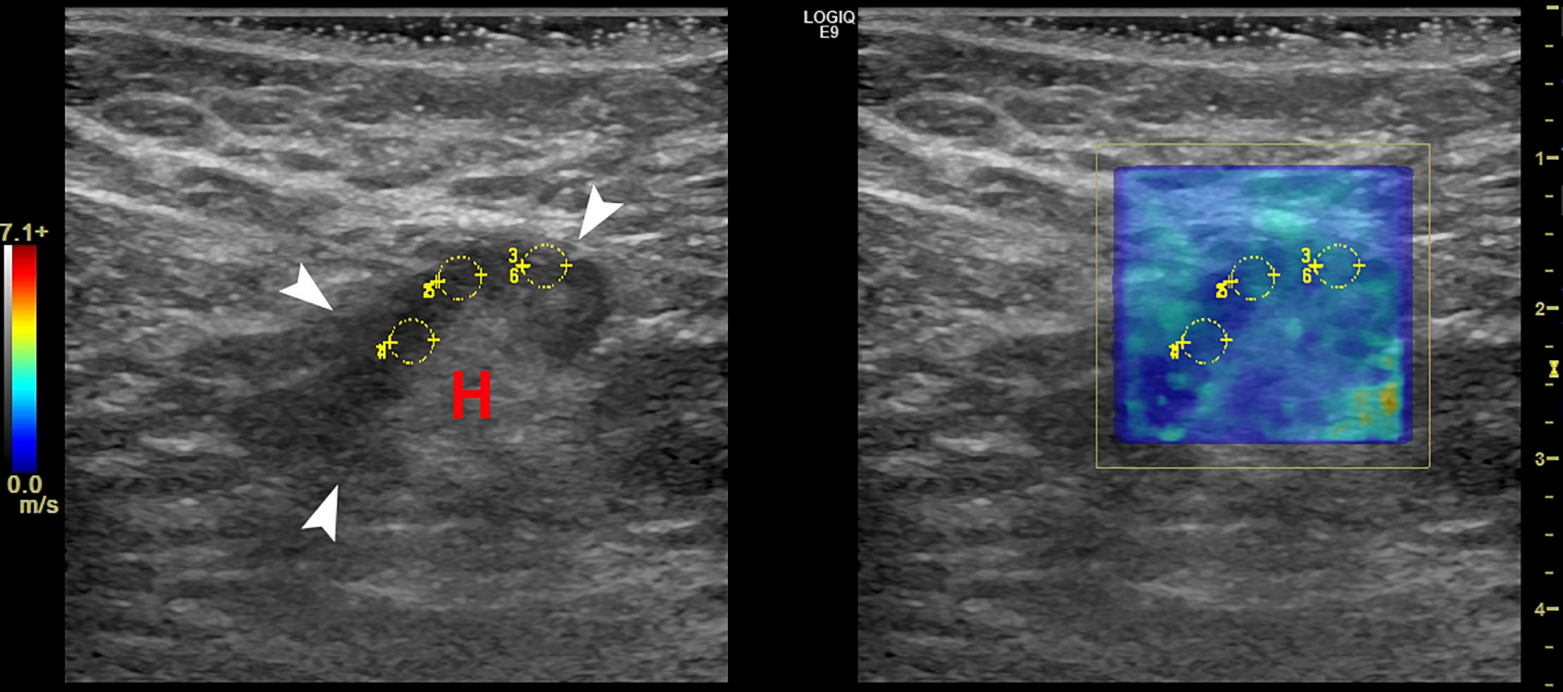
Left axillary B-mode (left) and shear wave speed map (right) images in a 45-year-old woman with invasive mammary carcinoma in the left breast. The US image shows 1 lymph node (white arrows) with mild cortical thickening measuring 4.6-mm and visible hilum (H). The elasticity results calculated from the average of the 3 ROIs (yellow) were E_mean_ = 10.1-kPa and E_max_ = 15.9-kPa. FNA biopsy results showed a mixed lymphoid population, most consistent with a reactive lymph node. Furthermore, the patient underwent SLN biopsy with negative results.

**Fig 5:**
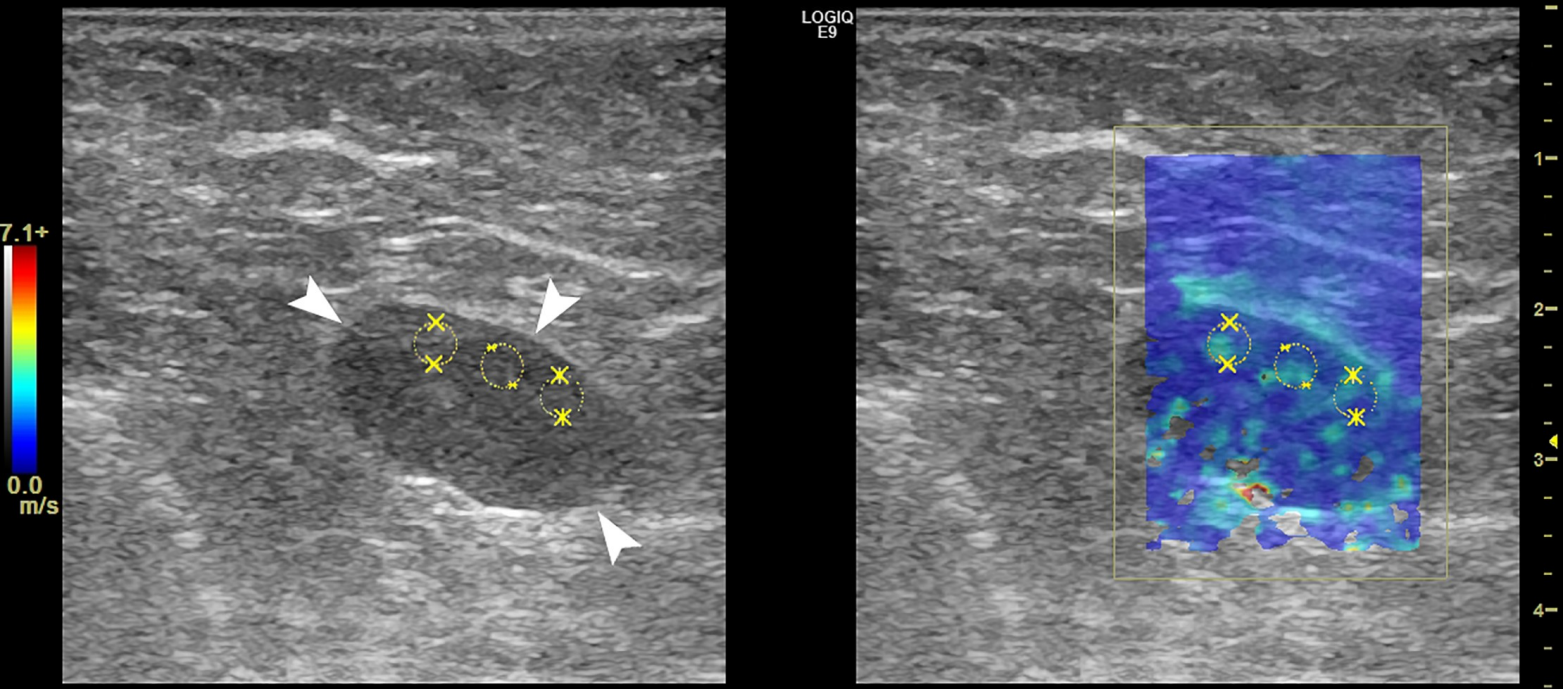
Left axillary B-mode (left) and shear wave speed map (right) images in a 60-year-old woman with a left breast mass and left axillary adenopathy identified on cardiac MRI. The US image shows an enlarged lymph node (white arrows) with a cortical thickness of 10.6-mm and absent hilum. The elasticity results calculated from the average of the 3 ROIs (yellow) were E_mean_ = 6.6-kPa and E_max_ = 20.1-kPa. FNA biopsy results showed a mixed lymphoid population consistent with a reactive intraparenchymal lymph node. Core needle biopsy of the breast mass resulted in a diagnosis of fibroadenoma with usual ductal hyperplasia.

**Fig 6:**
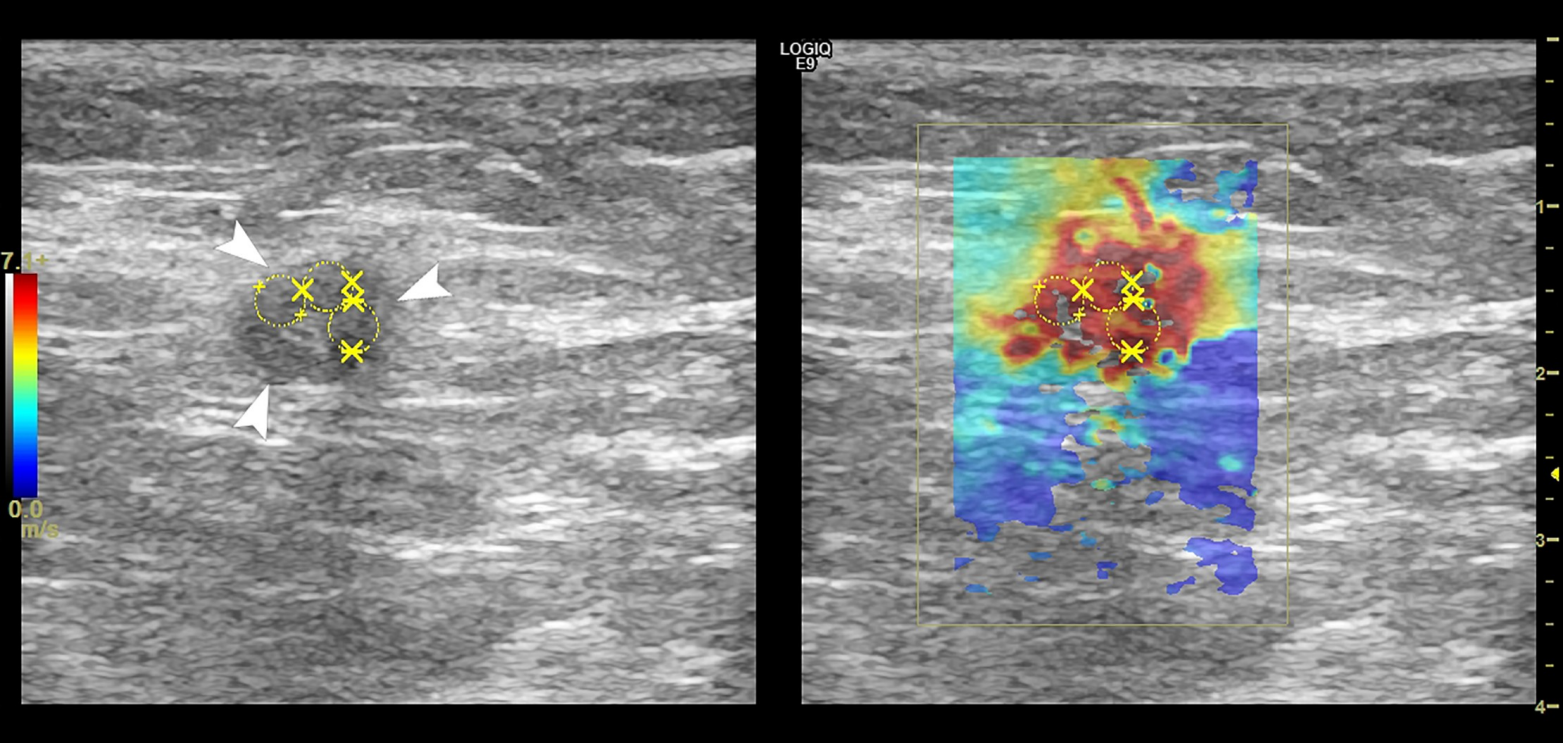
Left axillary US B-mode (left) and shear wave speed map (right) images in a 76-year-old woman with an infiltrating ductal carcinoma (grade II) in the left breast. The US image demonstrates a round, enlarged lymph node (white arrows) measuring 1.2 x 0.9 x 1.0-cm. The elasticity results calculated from the average of the 3 ROIs (yellow) were E_mean_ = 129.0-kPa and E_max_ = 268.7-kPa. FNA biopsy revealed metastatic adenocarcinoma involving fibroadipose tissue.

**Fig 7:**
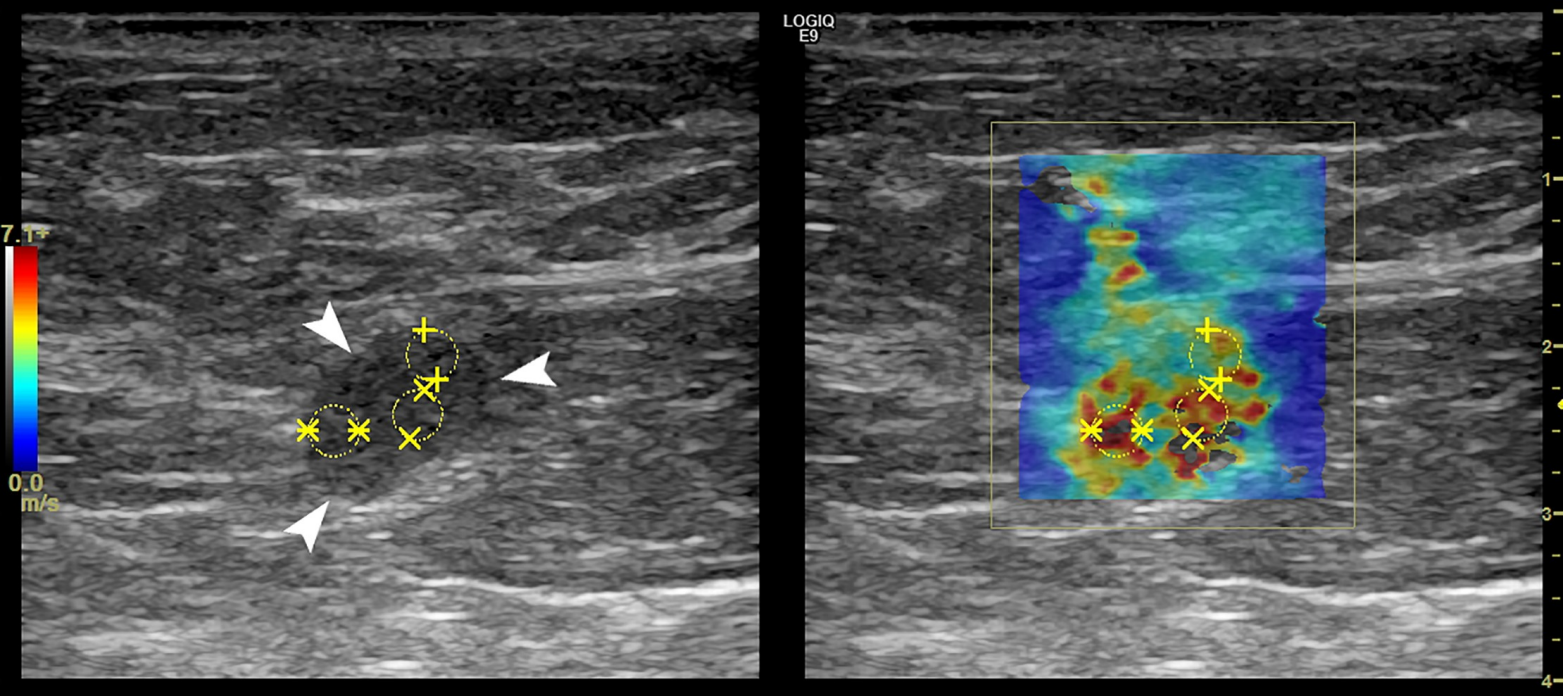
Right axillary B-mode (left) and shear wave speed map (right) images in a 78-year-old woman with a palpable mass in the right breast. The US image shows a level I lymph node (white arrows) demonstrating hilar effacement and cortical thickening (7.2-mm). The elasticity results calculated from the average of the 3 ROIs (yellow) were E_mean_ = 92.7-kPa and E_max_ = 170.1-kPa. FNA biopsy of the axillary lymph node was positive for adenocarcinoma. Right breast core needle biopsy demonstrated invasive ductal carcinoma, grade III.

**Fig 8:**
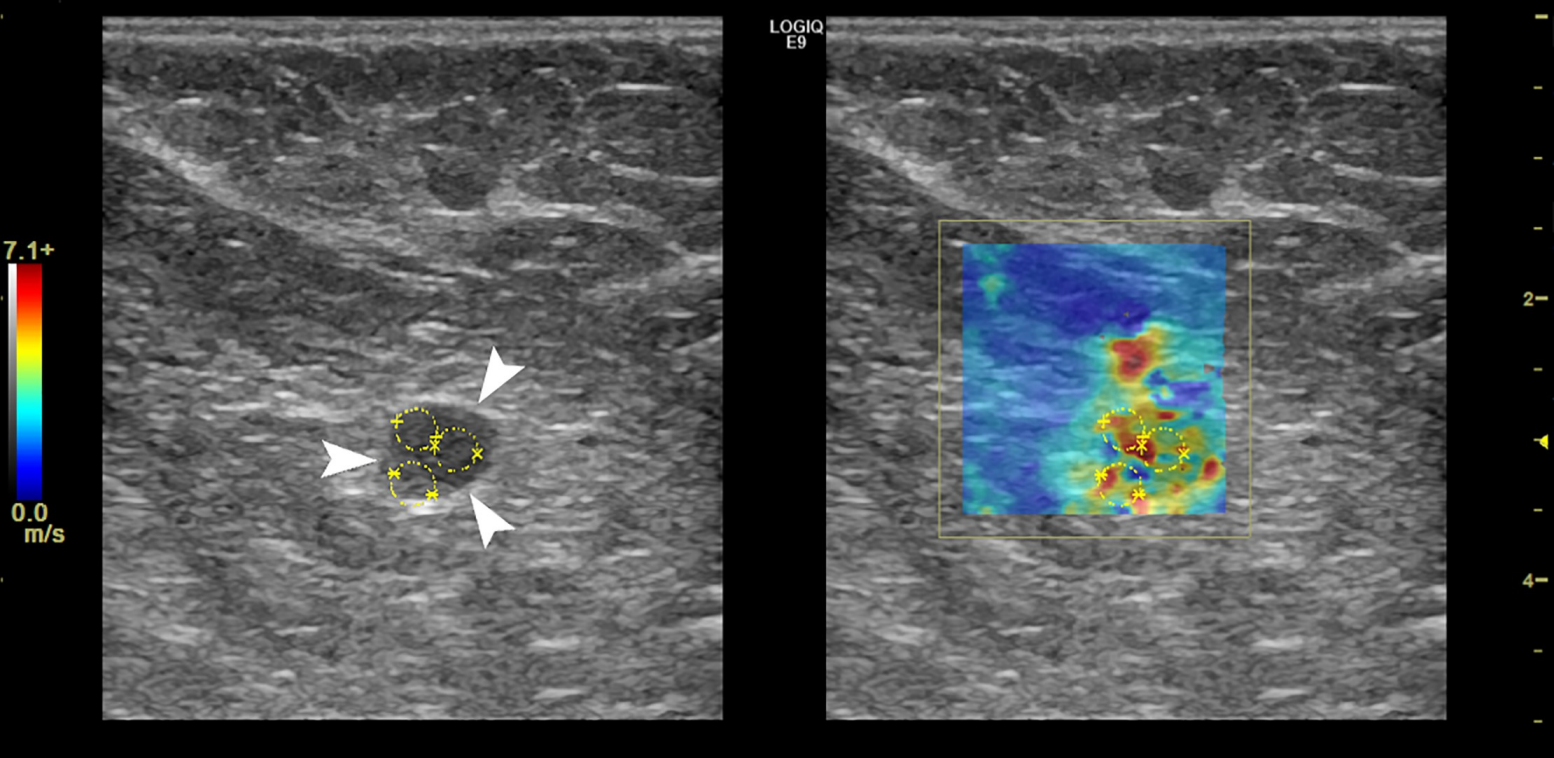
Right axillary B-mode (left) and shear wave speed map (right) images in a 61-year-old woman with invasive ductal carcinoma (grade II) in the right breast. The US image shows an abnormal, rounded lymph node (white arrows) with cortical thickening measuring 9-mm and absent fatty hilum. The elasticity results calculated from the average of the 3 ROIs (yellow) were E_mean_ = 77.9-kPa and E_max_ = 205.3-kPa. FNA biopsy result was positive for metastatic adenocarcinoma.

Figs 4,5 and Figs 6, 7 and 8 show the B-mode and elastography images of 5 different lymph nodes (2 reactive and 3 metastatic, respectively) demonstrating increased cortical thickening.

## Discussion

Ultrasonography is an important imaging tool in the assessment of abnormal axillary lymph nodes in breast cancer patients but is limited in its ability to differentiate reactive from metastatic ALNs [18]. CUSE provides tissue elasticity measurements with a unique excitation method that uses multiple simultaneous laterally spaced push beams [25]. The clinical utility of CUSE to differentiate between these two types of ALNs by elasticity has been demonstrated in this study. Metastatic ALNs have a higher elasticity when compared to reactive axillary lymph nodes because of the extracellular matrix changes due to the malignant process (i.e., increased collagen fiber density) [20]. The results from our study show that the Young’s modulus (i.e., measure of elasticity) from the lymph node cortex is significantly different when compared between reactive and malignant ALNs, distinguishing the two mechanisms with an accuracy of up to 92.3%.

Our results concur with those of previous investigations on lymph nodes performed with other SWE techniques, such as Supersonic Imagine [13, 14, 16, 26] and Virtual Touch Tissue Quantification [15, 27] in that malignant lymph nodes presented higher elasticity than benign lymph nodes. Most of these studies focused on the preoperative value of US SWE and primarily evaluated axillary SLNs as a result. Breast cancer patients can have one or more axillary SLNs, but these lymph nodes may not show morphological changes (i.e., normal lymph nodes). In the study [13] the average cortex thickness of benign lymph nodes was 1.88-mm, which falls within the thickness size of normal lymph nodes (<3-mm). Similarly, Kilic et. al [16] reported an average cortex thickness from benign lymph nodes of 1.6-mm. In our study, only patients with ALNs having a cortical thickness larger than 3mm were included. The average cortex thickness from reactive ALNs (i.e., benign) was 4.9-mm in our study population. Nonetheless, the mean elasticity values we obtained from the reactive ALNs were similar to those of normal ALNs found in the literature. The previously reported E_mean_ values ranged from 2.4 to 38.9-Kpa [13, 16, 26] compared to the E_mean_ values ranging between 2.4 and 39-kPa in our study. Regarding metastatic ALNs, our average E_mean_ and E_max_ value was 52.26-kPa and 126.6-kPa, respectively. These values are higher than those reported in the literature (average E_mean_ ranged from 17.47 to 50.2-kPa; average E_max_ ranged from 23.27 to 64.6-kPa) [13, 16, 26]. We believe this difference is caused by our study population as the metastatic ALNs presented severe cortical enlargement (mean size 7.8 mm) which is suggestive of a more developed lymph node invasion and thus higher cortex stiffness. Similarly, we observed higher cutoff values in our study (E_mean_ cutoff was 30.2-Kpa and the E_max_ cutoff was 66.5-Kpa), compared to the previously reported cutoffs (E_mean_ ranged from 14.75 to 20.79-Kpa and E_max_ from 24.67 to 25.8-Kpa) [13, 16, 26].

E_SD_ was the third best classification parameter showing that reactive ALNs present a more homogeneous elasticity map compared to metastatic ALNs. The combination of E_SD_ and E_mean_ results suggest that the morphological changes produced by the increase in number and size of follicular centers do not have an effect on the elastic properties of reactive ALNs. On the other hand, E_SD_ from metastatic ALNs presented a higher variance which is in agreement with the previous study by Youk et. al [13, 16, 26]. The higher variance is caused by the heterogeneity of the elasticity maps from metastatic ALNs which reflects the random and uncontrolled growth of cancer cells.

A major difference between this study and other studies performed with Supersonic Imagine was the ROI selection. Most studies [13, 16, 26] used a single 2-mm in diameter circular ROI on the stiffest area of the lymph node cortex; however, to reduce the subjectivity of selecting a small ROI area, we used multiple 3-mm non-overlapping circular ROIs to cover a larger cortex area. This approach resulted in a similar performance compared to E_max_. More objectively, Tan et.al [13, 14, 16, 26] obtained the elastic measurements by manually drawing the lymph node contour on the B-mode image using the Supersonic trace tool, a feature not available in LE9.

One of the limitations of our study is the comparison of elasticity parameters to FNA biopsy results. The sensitivity of ALN FNA biopsy in breast cancer patients reportedly ranges from 65% to 79.4% [28–31]. In our study 29 of 38 patients (76%) with breast cancer and negative axillary FNA biopsy results underwent SLN biopsy with benign findings. The remaining 9 patients were yet to complete the rest of the workup and did not have SLN biopsy results, so metastasis or micrometastasis could not be completely ruled out. From this group, 3 lymph nodes were false positive cases based on increased mean elasticity and negative axillary FNA biopsy. Another limitation is when only a few cancer cells are present in an ALN and the early changes on the extracellular matrix may not show a significant increase on the lymph node elasticity compared to a normal/reactive ALN, resulting in a false negative SWE finding. In our study the average cortical thickness of all false negative SWE cases (n = 8) was 4.8-mm, with mostly no cortical lobulation or absence of fatty hilum, which indicate early changes [5].

A third limitation was the low signal to noise ratio of shear waves when imaging deep ALNs. In our study, 3 ALNs were located below 3 cm, where most of the elastogram reconstruction was on the upper region of the ALN. For these cases, the measurements were obtained from the upper lymph node area within the cortex. Lastly, the anisotropy of tissues around ALNs was not considered. Anisotropic tissues, such as muscle, can increase or decrease the speed of shear waves depending on the orientation of the transducer [32]. In the axillary area, the pectoralis minor muscle may have influenced some of the ALN elasticity measurements. Only elasticity maps from the largest cortex cross-section were taken into account for the statistical analysis.

In conclusion, CUSE provides a quantifiable parameter that could be used for the assessment of abnormal axillary lymph nodes to differentiate reactive and metastatic processes. This noninvasive technique can be beneficial when assessing ALNs targeted for FNA or SLN biopsy.
